# Current Status and influencing factors of adolescents’ awareness of functional gastrointestinal disorders and health education needs: a nationwide cross-sectional study

**DOI:** 10.3389/fped.2026.1805032

**Published:** 2026-04-20

**Authors:** Ming Shen, Mingfang Wei, Wei Wang, Hua Lan, Di Xia, Ming-guang Zhang, Changqing Liu, Hongfei Ren

**Affiliations:** 1 Department of Gastroenterology, West China Hospital/West China School of Nursing, Sichuan University Chengdu, Sichuan, China; 2 Trauma Center of West China Hospital, West China School of Nursing, Sichuan University, Chengdu, Sichuan, China

**Keywords:** adolescent, awareness, cross-sectional studies, functional gastrointestinal disorders, health education

## Abstract

Functional gastrointestinal disorders (FGIDs) are prevalent among teenagers, yet understanding of them is limited. This study aimed to evaluate adolescents’ awareness of FGIDs, identify factors influencing this awareness, and assess adolescents’ health education needs, thereby providing evidence for targeted health education strategies. A nationwide cross-sectional survey conducted from June to August 2024 assessed 1,612 adolescents aged 10–19 years. Despite the survey's broad scope, only 4.7% exhibited a systematic understanding of FGIDs, and merely 3.7% reported high familiarity, underscoring a pronounced knowledge deficit in this population. The majority of respondents (39.8%) were only familiar with it, while 25% had not heard of it. Marked disparities in awareness were identified based on growth environment, level of education, boarding status, caregiver type, personality traits, participation in social activities, and the experience of FGIDs by respondents or their contacts (*P* < 0.05). The results demonstrate that teenagers typically possess limited awareness of FGIDs. It is advisable to formulate tailored health education tactics based on age, educational attainment, and living conditions to enhance knowledge dissemination, deepen comprehension, and elevate adolescents’ disease awareness and self-management skills.

## 1 Introduction

Functional gastrointestinal disorders (FGIDs) significantly impact a considerable number of teenagers worldwide, presenting as chronic or recurrent symptoms such as abdominal discomfort, bloating, constipation, and diarrhea without any structural pathology (1). In a multicountry pediatric survey, approximately 23.1% of children and adolescents satisfied the criteria for at least one FGID (2). These illnesses can adversely affect physical health, emotional well-being, academic achievement, and social functioning in adolescents (1). In China, a study in southern Anhui Province reported a 14.3% prevalence of functional abdominal pain disorders among children aged 6–17 years. FGIDs significantly impact not only the physical health of adolescents but also their mental well-being, academic performance, and social interactions, leading to substantial psychological distress and a reduction in quality of life (3). Due to their widespread occurrence and considerable psychosocial impact, FGIDs in adolescents have emerged as a critical global public health concern that necessitates heightened awareness and timely intervention.

Notwithstanding the significant disease burden, awareness and comprehension of FGIDs among adolescents remain constrained. The majority of current research emphasizes symptom epidemiology and risk factors, rather than cognitive awareness or health education (4). For instance, a prevalence study in India found that 15.85% of adolescents suffered from FGIDs, with functional abdominal pain disorders being the most common (4, 5). In China, university-based studies applying the Rome IV criteria have examined the prevalence of functional dyspepsia and irritable bowel syndrome (6). Nevertheless, limited research has investigated adolescent populations or the factors influencing disease awareness. Furthermore, current studies have predominantly focused on prevalence, psychological comorbidities, or nutritional elements such as hydration and boarding status, but investigations into the unique educational requirements of teenagers and their associated factors are few (7, 8). Studies have shown that many adolescents fail to recognize the symptoms of FGIDs or understand the potential causes and treatments (9). International research indicates that adolescents often lack sufficient knowledge about FGID management and prevention, with many not seeking medical help until symptoms become severe (9). In China, studies show that while the overall prevalence of FGIDs in adolescents is similar to that in Western countries, awareness and education on the topic are considerably lower, with only a small proportion of adolescents receiving adequate health education (10).

This study posits that teenagers’ awareness of FGIDs is typically minimal and shaped by sociodemographic, contextual, and experiential factors. This study seeks to (1) evaluate the existing degree of FGID knowledge among adolescents, (2) identify the primary drivers influencing awareness, and (3) elucidate adolescents’ health education requirements. The findings will yield evidence-based insights for formulating age-appropriate, context-sensitive, and targeted health education initiatives aimed at enhancing disease understanding, early detection, and self-management within this demographic.

## 2 Subjects and methods

### 2.1 Study design

This study was a nationwide cross-sectional survey conducted from August to November 2024, aimed at assessing adolescents’ awareness of FGIDs and identifying influencing factors.

### 2.2 Participants

Adolescents aged 10–19 years from across China were recruited as study participants.

**Inclusion criteria:** (1) aged between 10 and 19 years; (2) voluntarily participated and completed the questionnaire.

**Exclusion criteria:** (1) individuals diagnosed with diabetes, hyperthyroidism or hypothyroidism, peptic ulcer, inflammatory bowel disease, gastrointestinal tumors, or other severe medical or surgical conditions; (2) a history of abdominal surgery; (3) recent symptoms such as melena, hematochezia, hematemesis, abnormal anemia, fever, unexplained weight loss, altered bowel habits, or dysphagia; (4) use of medications that could affect gastrointestinal symptom assessment within the past four weeks, such as anti-inflammatory analgesics, sedatives, anti-anxiety agents, or antidepressants.

The sample size was calculated based on the rule of thumb that the minimum sample size should be 10 times the number of questionnaire items, resulting in a required minimum of 552 participants.

### 2.3 Measures

The survey included a structured questionnaire designed to collect data on participants’ demographic information, living environment, health status, awareness of FGIDs, and related health education needs. The questionnaire was developed based on a literature review and expert consultation to ensure content validity and clarity.

### 2.4 Data collection

Data were collected through an online questionnaire distributed via *Wenjuanxing* (Questionnaire Star). The survey link was disseminated through social media platforms, youth organizations, and school networks to ensure wide geographical coverage. Respondents could complete the questionnaire anonymously using electronic devices.

### 2.5 Data analysis

All data were entered into Excel and analyzed using SPSS version 25.0 (IBM Corp., Armonk, NY, USA). Categorical variables were described using frequencies and percentages. Continuous variables were presented as medians and interquartile ranges if non-normally distributed. Chi-square tests or rank-sum tests were used for group comparisons. Spearman correlation analysis was employed to assess relationships between variables, and logistic regression was used for multivariate analysis. A two-sided *p-value* < 0.05 was considered statistically significant.

### 2.6 Ethical considerations

Ethical approval for this study was obtained from the Institutional Ethics Committee (approval number to be provided after review). Participation was voluntary, and informed consent was obtained from all respondents prior to data collection. For participants under 18 years old, consent was obtained from their guardians.

This study was approved by the Ethics Committee of West China Hospital, Sichuan University, with a review number: 2025 Annual review (1054).

## 4 Results

A total of 1,686 questionnaires were collected from the adolescent population in this survey. After excluding 74 invalid questionnaires, 1,612 valid questionnaires were ultimately obtained, with an effective response rate of 96.61%.

### 4.1 Basic characteristics

The age distribution of the 1,612 adolescents ranged from 10 to 19 years, with a positively skewed distribution. The median age was 17.00, and the interquartile range (*IQR*) was 14.00–18.00. Other basic characteristics are presented in Tables 1, 2.

**Table 1:** Basic demographic characteristics of the adolescent population (*n* = 1,612).

Characteristics	Group	Number	Percentage (%)	U/H	*P* value
Sex	Male	531	32.94	U = 2,71,286.00	0.06
	Female	1,081	67.06		
Age	–	1,612	–	H = 61.11	<0.001
Ethnic group	Han Chinese	1,198	74.32	H = 13.33	<0.001
	Tibetan	13	0.81		
	Others	401	24.88		
Early-life environment	City	404	25.06	H = 21.66	<0.001
	County seat	505	31.33		
	Rural area	703	43.61		
Habitat	Southwest region	601	37.28	H = 19.831	<0.001
	Northwestern region	174	10.79		
	Central China	530	32.88		
	North China region	157	9.74		
	Other regions	150	9.31		
Educational attainment	Primary school	113	7.01	H = 57.027	<0.001
	Junior high school	496	30.77		
	Senior high school	364	22.58		
	Associate degree/Bachelor's degree	639	39.64		
Boarding student	Yes	891	55.27	U = 2,71,936.50	<0.001
	No	721	44.73		
Singleton	Yes	374	23.2	U = 2,28,910.50	0.73
	No	1,238	76.8		
Primary caregiver	Parents	1,172	72.7	H = 10.11	0.02
	Single-parent family	216	13.4		
	Grandparents	200	12.41		
	Others	24	1.49		
Family structure	Nuclear family	1,019	63.21	H = 6.31	0.1
	Extended family	342	21.22		
	Single-parent family	164	10.17		
	Blended family	87	5.4		
Body mass index (BMI)	Underweight	233	14.45	H = 7.42	0.06
	Normal	1,059	65.69		
	Overweight	170	10.55		
	Obesity	150	9.31		

**Table 2:** Psychosocial and FGID-related characteristics of the adolescent population (*n* = 1,612).

Characteristics	Group	Number	Percentage (%)	U/H	*P* value
Personality	Introverted	96	5.96	H = 11.44	0.02
	Slightly introverted	330	20.47		
	Neutral	676	41.94		
	Slightly extroverted	348	21.59		
	Extroverted	162	10.05		
Social activities	Strongly dislike	77	4.78	H = 13.94	0.01
	Dislike	266	16.5		
	Neutral	689	42.74		
	Like	325	20.16		
	Strongly like	255	15.82		
Have people around you (excluding yourself) ever been diagnosed with FGIDs?	Yes	295	18.3	U = 1,52,611.50	<0.001
	No	1,317	81.7		
Have you yourself ever been diagnosed with FGIDs?	Yes	116	7.2	U = 62,279.50	<0.001
	No	1,496	92.8		
Are you familiar with functional gastrointestinal disorders?	Never	403	25	–	–
	Only aware of the existence	642	39.83		
	Some knowledge	433	26.86		
	Systematic understanding	75	4.65		
	Very familiar	59	3.66		

### 4.2 Current status of adolescents’ awareness of FGIDs

Among the 1,612 adolescents surveyed, awareness of functional gastrointestinal disorders (FGIDs) was predominantly minimal. Only 4.7% (*n* = 75) possessed a thorough comprehension of the disorder, and 3.7% (*n* = 59) were highly knowledgeable about it, whilst the rest exhibited minimal awareness—39.8% (*n* = 642) had merely heard of FGIDs, and 25.0% (*n* = 403) were entirely unfamiliar with them. Marked disparities in awareness levels were noted across various characteristics, including educational attainment, residential setting, boarding status, primary caregiver, personality traits, engagement in social activities, and the experience of FGIDs by respondents or their acquaintances (*P* < 0.05). The correlation analysis between awareness level and each variable is shown in Table 3.

**Table 3:** Correlation analysis of factors associated with adolescent awareness levels (*n* = 1,612).

Characteristics	Are you familiar with functional gastrointestinal disorders
Sex	0.06
Age	**<0**.001**
Ethnic group	**<0**.001**
Early-life environment	**0**.01*
Habitat	0.39
Educational attainment	**<0**.001**
Boarding student	**<0**.001**
Singleton	0.73
Primary caregiver	0.12
Family structure	0.06
Body mass index (BMI)	0.40
Personality	**0**.01*
Social activities	**<0**.001**
Have people around you (excluding yourself) ever been diagnosed with functional gastrointestinal disorders in the past or currently	**<0**.001**
Have you yourself ever been diagnosed with functional gastrointestinal disorders in the past or currently?	**<0**.001**

*Correlation is significant at the 0.05 level (2-tailed).

**Correlation is significant at the 0.01 level (2-tailed).

Bold values indicate statistically significant results (*P* < 0.05).

### 4.3 Influencing factors of adolescents’ awareness of FGIDs

Age and ethnicity demonstrated no substantial correlation with awareness and were omitted from the final model. The regression analysis (Table 4) indicated the absence of multicollinearity among independent variables and satisfied the parallel lines assumption (*χ*^2^ = 93.34, *P* = 0.11).

**Table 4:** The results of the ordered logistic regression analysis of adolescents’ cognitive levels (*n* = 1,612).

Characteristics	β	SE	Waldc^2^	*P*	95%CI	OR
Early-life environment						
City	−0.04	0.14	0.09	0.76	−0.31∼0.22	0.96
County seat	0.32	0.12	7.49	**0**.**01**	0.09∼0.55	1.38
Rural area	-	-	-	-	-	-
Habitat						
Southwest region	−0.12	0.18	0.45	0.50	−0.47∼0.23	0.89
Northwestern region	0.36	0.23	2.51	0.11	−0.09∼0.80	1.43
Central China	0.80	0.20	15.75	**<0**.**001**	0.40∼1.19	2.22
North China region	−0.55	0.23	5.75	**0**.**02**	−0.99∼−0.10	0.58
Other regions	-	-	-	-	-	-
Educational attainment						
Primary school	−1.33	0.25	27.60	**<0**.**001**	−1.82∼−0.83	0.27
Junior high school	−0.40	0.16	5.99	**0**.**01**	−0.72∼−0.08	0.67
Senior high school	−0.59	0.17	12.75	**0**.**00**	−0.92∼−0.27	0.55
Associate degree/Bachelor's degree	-	-	-	-	-	-
Boarding student						
Yes	0.73	0.15	25.32	**<0**.**001**	0.45∼1.01	2.08
No	-	-	-	-	-	-
Primary caregiver						
Parents	−0.99	0.38	6.69	**0**.**01**	−1.74∼−0.24	0.37
Single-parent family	−1.17	0.40	8.61	**0**.**00**	−1.95∼−0.39	0.31
Grandparents	−1.04	0.40	6.76	**0**.**01**	−1.83∼−0.26	0.35
Others	-	-	-	-	-	-
Personality						
Introverted	−0.33	0.27	1.46	0.23	−0.87∼0.21	0.72
Slightly introverted	−0.52	0.21	6.10	**0**.**01**	−0.93∼−0.11	0.59
Neutral	−0.29	0.20	2.19	0.14	−0.67∼0.09	0.75
Slightly extroverted	−0.33	0.20	2.84	0.09	−0.72∼0.05	0.72
Extroverted	-	-	-	-	-	-
Social activities						
Strongly dislike	−0.83	0.28	9.03	**0**.**00**	−1.37∼−0.29	0.44
Dislike	−0.60	0.19	9.66	**0**.**00**	-.98∼−0.22	0.55
Neutral	−0.45	0.17	7.39	**0**.**01**	-.78∼−0.13	0.64
Like	−0.20	0.18	1.33	0.25	-.55∼0.14	0.82
Strongly like	-	-	-	-	-	-
Have people around you (excluding yourself) ever been diagnosed with functional gastrointestinal disorders in the past or currently						
Yes	0.65	0.13	23.52	**<0**.**001**	0.39∼0.91	1.92
No	-	-	-	-	-	-
Have you yourself ever been diagnosed with functional gastrointestinal disorders in the past or currently						
Yes	0.841	0.199	17.863	**<0**.**001**	0.451∼1.232	2.32
No	-	-	-	-	-	-

Bold values indicate statistically significant results (*P* < 0.05).

## 5 Discussion

### 5.1 Current status of adolescents’ awareness of FGIDs

This study aimed to investigate adolescents’ awareness of FGIDs, identify the factors influencing this awareness, and explore their health education needs. The results revealed that most adolescents were only aware of the existence of such diseases (39.83%), with 25.00% having never heard of them and 26.86% having some knowledge of their definition and etiology. Only a small proportion demonstrated systematic understanding (4.65%) or comprehensive knowledge (3.66%).

The findings indicate significant structural deficiencies in Chinese adolescents’ cognition of FGIDs, primarily manifested in two dimensions: inadequate coverage of fundamental knowledge and a severe lack of in-depth understanding. Specifically, approximately one-quarter of respondents (25.00%) showed complete cognitive blankness - unable to even recognize the basic concept of FGIDs - reflecting substantial gaps in basic health education coverage. Moreover, merely 8.31% of adolescents achieved a systematic understanding, with fewer than 4% comprehending disease mechanisms. This “pyramid-shaped” cognitive structure exposes the failure of current health education in constructing profound knowledge frameworks.

This cognitive status sharply contradicts the requirement for long-term health management of FGIDs, necessitating targeted improvement through stratified educational strategies (11). These should not only expand universal coverage of basic concepts but also enhance cognitive depth through case-based teaching and interactive courses (8).

### 5.2 Influencing factors of adolescents’ awareness of FGIDs

#### 5.2.1 Early-life environment and habitat

Variations in early living environments may lead to disparities in adolescents’ FGIDs awareness, primarily due to differences in healthcare resources, health consciousness, socioeconomic status, environmental factors, and information accessibility (12). Urban areas typically benefit from better healthcare infrastructure, comprehensive health education, and greater health information exposure, whereas rural regions often face limited medical resources and insufficient health literacy, resulting in poorer digestive health awareness. Urban residents generally exhibit higher health consciousness, greater educational attainment, and more proactive health-seeking behaviors, while rural populations may prioritize immediate livelihood concerns over preventive healthcare (13). Economic disparities further exacerbate this gap, as urban households can afford medical expenses and health investments more readily than their rural counterparts. Environmental factors also play a role—urban stressors (e.g., pollution, fast-paced lifestyles) may increase FGIDs awareness due to higher prevalence, whereas rural areas, with slower lifestyles and cleaner environments, may perceive less urgency in digestive health education. Additionally, urban settings facilitate faster health information dissemination and stronger social support systems, while rural communities often experience delayed or insufficient health communication (14).

Provincial differences in FGIDs awareness among adolescents are similarly influenced by regional healthcare access, health education policies, dietary habits, sociocultural attitudes, economic conditions, and media reach (15). Areas with robust medical systems and structured school-based health programs foster better disease awareness, while socioeconomic and cultural factors shape health priorities and information-seeking behaviors (14).

The observed cognitive disparities reflect systemic inequities in health resource allocation and education accessibility. To bridge this gap, targeted interventions—such as enhanced rural health education, equitable resource distribution, and improved health communication strategies—are essential to promote health equity and reduce urban-rural disparities in FGIDs awareness.

#### 5.2.2 Educational attainment

Adolescents at different educational stages exhibit differences in health education regarding Functional Gastrointestinal Disorders (FGIDs). These differences are primarily reflected in the frequency and depth of educational activities (16). During primary school, health education mainly focuses on basic hygiene habits, with lower frequency and shallower depth of teaching activities. In junior high school, systematic physiological health knowledge is introduced, but the teaching remains at the level of symptom description, with increased frequency but still limited depth. In senior high school, as students’ cognitive abilities improve, health education begins to cover more in-depth content such as disease mechanisms, though the actual frequency of implementation may decrease (17). At the university stage, conditions are in place for specialized and systematic health education, with both frequency and depth reaching higher levels. These stage-specific differences in educational resource allocation may lead to varying levels of FGIDs knowledge among adolescents with different educational backgrounds.

#### 5.2.3 Primary caregiver

The education level of caregivers and family structure significantly impact adolescents’ understanding of Functional Gastrointestinal Disorders (18). Caregivers with higher education levels typically possess stronger abilities to acquire and disseminate knowledge. They can better comprehend health information and convey it to their children in a scientific manner, which helps enhance the children's knowledge of FGIDs. Conversely, caregivers with lower education levels may have limited understanding of FGIDs themselves and struggle to accurately pass on relevant information, resulting in less accurate or in-depth disease knowledge among children. In intact families, where both parents are involved in their children's upbringing and education, a more comprehensive focus on the children's health, including FGIDs education, is likely. However, in single-parent or extended-family caregiving situations, caregivers may have limited energy or a narrower knowledge base, leading to less comprehensive health education for the children and thus affecting their understanding of FGIDs (7).

#### 5.2.4 Boarding student

Boarding students, through systematic school health education, regulated dietary management, and abundant peer interactions, typically gain more comprehensive and accurate health knowledge, resulting in a relatively higher level of understanding of Functional Gastrointestinal Disorders. On the other hand, non-boarding students rely more on their family environment for health information, and their level of understanding may vary significantly depending on the family's health awareness and dietary habits (19). Therefore, schools and families should work together to provide rich health education resources for adolescents. Whether boarding or not, attention should be paid to adolescents’ knowledge of Functional Gastrointestinal Disorders and their health education needs.

#### 5.2.5 Social activities and personality

Both personality and participation in social activities significantly influence adolescents’ understanding of Functional Gastrointestinal Disorders. Teenagers with an open personality are more willing to actively explore and learn related knowledge (20). Extroverted adolescents obtain information through social interactions. Social activities provide adolescents with a platform for information sharing, knowledge dissemination, and social support. In these activities, adolescents can acquire more comprehensive and accurate health knowledge by interacting with peers, teachers, and health experts. For example, school-organized health lectures, community health activities, or online health forums can help them understand the symptoms, causes, and treatments of Functional Gastrointestinal Disorders. Moreover, behavioral imitation and health education in social activities help adolescents develop good living habits, reduce anxiety and stress, and thus better manage their health (20). Therefore, when conducting health education, schools and families should consider adolescents’ personality traits and adopt personalized educational strategies. At the same time, they should encourage adolescents to actively participate in social activities and ensure that these activities provide high-quality health information and professional guidance to enhance adolescents’ knowledge and management capabilities regarding Functional Gastrointestinal Disorders.

#### 5.2.6 Having experienced illness

The experience of illness, either personally or through someone close, significantly impacts adolescents’ understanding of Functional Gastrointestinal Disorders. Direct experience and contact with medical professionals provide them with more accurate health knowledge, and the process of self-management enhances their health awareness (21).

### 5.3 Strengths and limitations

This study has several strengths. First, it used a nationwide cross-sectional design with a relatively large sample size, which enhances the representativeness of the findings. Second, the study explored not only adolescents’ awareness of functional gastrointestinal disorders (FGIDs) but also the factors influencing awareness and their health education needs, providing a comprehensive understanding of the issue. Third, the use of a structured questionnaire and multivariate statistical analysis improved the reliability of the findings. However, several limitations should also be acknowledged. As a cross-sectional study, causal relationships between variables cannot be established. In addition, the data were collected through self-reported online questionnaires, which may be subject to recall bias or reporting bias. Furthermore, although participants were recruited nationwide, the sample may not fully represent all adolescents in China. Future studies could employ longitudinal designs and more diverse sampling strategies to further validate and expand these findings.

### 5.4 Future research directions

Future research should further explore adolescents’ awareness and management of FGIDs using longitudinal study designs to better understand causal relationships between influencing factors and awareness levels. In addition, future studies could include more diverse regional samples and incorporate qualitative methods to gain deeper insights into adolescents’ perceptions, experiences, and educational needs related to FGIDs. Developing and evaluating targeted health education interventions for adolescents in schools and communities would also be valuable for improving disease awareness and promoting effective self-management.

## 6 Conclusion

This study found that adolescents generally have a low level of awareness of FGIDs, with only a small proportion demonstrating systematic or comprehensive knowledge. Awareness levels were significantly associated with several factors, including early-life environment, educational attainment, boarding status, primary caregiver, personality traits, participation in social activities, and personal or surrounding experiences with FGIDs. These findings highlight substantial gaps in adolescents’ knowledge of FGIDs and emphasize the need for targeted health education programs. Tailored educational strategies based on educational stage, living environment, and family context may help improve adolescents’ understanding of FGIDs and promote better health awareness and self-management.

## Data Availability

The original contributions presented in the study are included in the article/supplementary material, further inquiries can be directed to the corresponding authors.
