# Correction: Current status and influencing factors of adolescents’ awareness of functional gastrointestinal disorders and health education needs: a nationwide cross-sectional study

**DOI:** 10.3389/fped.2026.1892151

**Published:** 2026-06-19

**Authors:** Ming Shen, Mingfang Wei, Wei Wang, Hua Lan, Di Xia, Ming-Guang Zhang, Changqing Liu, Hongfei Ren

**Affiliations:** 1 Department of Gastroenterology, West China Hospital/West China School of Nursing, Sichuan University, Chengdu, Sichuan, China; 2 Trauma Center of West China Hospital, West China School of Nursing, Sichuan University, Chengdu, Sichuan, China

**Keywords:** adolescent, awareness, cross-sectional studies, functional gastrointestinal disorders, health education

## Funding

In the published article, there was an error in the Funding statement. The correct Funding statement is below.

The author(s) declared that financial support was received for this work and/or its publication. This study was supported by the Sichuan Province Science and Technology Support Program (Grant no. 2024JDKP0097).

The original article has been updated.

